# Biocompatible Porous Polyester-Ether Hydrogel Scaffolds with Cross-Linker Mediated Biodegradation and Mechanical Properties for Tissue Augmentation

**DOI:** 10.3390/polym10020179

**Published:** 2018-02-12

**Authors:** Berkay Ozcelik, Jason Palmer, Katharina Ladewig, Paula Facal Marina, Geoffrey W. Stevens, Keren Abberton, Wayne A. Morrison, Anton Blencowe, Greg G. Qiao

**Affiliations:** 1 Department of Chemical & Biomolecular Engineering, The University of Melbourne, Parkville, Melbourne, VIC 3010, Australia; ozcelikb@unimelb.edu.au (B.O.); kladewig@unimelb.edu.au (K.L.); gstevens@unimelb.edu.au (G.W.S.); 2 O’Brien Institute Department, St. Vincent’s Institute of Medical Research, Melbourne, VIC 3065, Australia; jpalmer@svi.edu.au (J.P.); abberton@unimelb.edu.au (K.A.); wayne.morrison@unimelb.edu.au (W.A.M.); 3 School of Pharmacy and Medical Sciences, University of South Australia, Adelaide, SA 5000, Australia; Paula.FacalMarina@unisa.edu.au; 4 Future Industries Institute, University of South Australia, Mawson Lakes, SA 5095, Australia

**Keywords:** polyester-ether, hydrogel, scaffold, biocompatible, biodegradation

## Abstract

Porous polyester-ether hydrogel scaffolds (PEHs) were fabricated using acid chloride/alcohol chemistry and a salt templating approach. The PEHs were produced from readily available and cheap commercial reagents via the reaction of hydroxyl terminated poly(ethylene glycol) (PEG) derivatives with sebacoyl, succinyl, or trimesoyl chloride to afford ester cross-links between the PEG chains. Through variation of the acid chloride cross-linkers used in the synthesis and the incorporation of a hydrophobic modifier (poly(caprolactone) (PCL)), it was possible to tune the degradation rates and mechanical properties of the resulting hydrogels. Several of the hydrogel formulations displayed exceptional mechanical properties, remaining elastic without fracture at compressive strains of up to 80%, whilst still displaying degradation over a period of weeks to months. A subcutaneous rat model was used to study the scaffolds in vivo and revealed that the PEHs were infiltrated with well vascularised tissue within two weeks and had undergone significant degradation in 16 weeks without any signs of toxicity. Histological evaluation for immune responses revealed that the PEHs incite only a minor inflammatory response that is reduced over 16 weeks with no evidence of adverse effects.

## 1. Introduction

3D scaffolds with appropriately engineered characteristics (e.g., mechanical properties, biodegradability, and tissue-material interactions) for the regeneration of tissues are central to the concept of tissue engineering. Regardless of whether the scaffolds are cultured in vitro with cells and then implanted into an injured site, or are implanted directly into the injured site to promote tissue regeneration in vivo from the host system, the requirements and challenges of scaffold engineering are well recognised [1,2,3]. For soft-tissue engineering, hydrogel scaffolds have displayed great promise as a result of their unique compositional and structural similarities that mimic those of the natural extracellular matrix (ECM) and provide a desirable framework for the proliferation and survival of cells. Nevertheless, both physicochemical and classical mechanical parameters, including the biodegradation, porosity, and surface chemistry, as well as biological performance parameters—biocompatibility and cell adhesion—must be tuned for specific applications. One of the major challenges is the optimization of all of these parameters in a single formulation. For example, improvements in the mechanical properties are generally at the detriment of the degradability. In addition, the accessibility and commercial feasibility also needs to be considered when developing scaffolds. Therefore, finding an optimal biomaterial that combines all the necessary characteristics for tissue regeneration remains a major objective of contemporary tissue engineering.

A wide range of natural and synthetic materials have been used to fabricate hydrogel scaffolds, as well as combinations thereof. In particular, poly(ethylene glycol) (PEG) derivatives have been extensively studied as they can be fabricated into hydrogels with tailorable mechanical and swelling properties matching various tissues types [4], and are generally considered bio-inert (i.e., non-immunogenic and anti-protein fouling). While the latter is not necessarily conducive to cell adhesion and tissue formation, various strategies have been reported to conjugate bioactive molecules to modulate specific cellular responses [5]. Numerous synthetic approaches have been devised for the preparation of covalently cross-linked PEG-based hydrogels [6] including radical polymerisation of PEG di(meth)acrylate macromonomers [7], Michael-type additions [8,9,10], and epoxy-amine [11], hydrazide-aldehyde [12], thiol-ene [13,14,15] and azide-alkyne chemistries [16,17] to mention a few. Furthermore, these approaches generally allow hydrolytically or enzymatically degradable components to be incorporated to afford biodegradable PEG-based hydrogels. Common approaches involve the modification of PEG derivatives with hydrolytically susceptible polyester and polycarbonate segments [10,13,14,18,19,20], or the use of short peptide cross-linkers that can be degraded by specific enzymes [8,9,21]. 

Evidently, the development of sophisticated PEG-based hydrogel systems—such as those described above—requires the synthesis and purification of suitable PEG precursors, cross-linkers and bioactive components to provide desirable physicochemical properties and cellular interactions. In general, these components need to be custom prepared, or if available commercially, are expensive. Thus, there remains significant interest in the development of PEG-based hydrogels with suitable tissue engineering characteristics from cheap and readily available commercial reagents without complex manufacturing steps. Furthermore, simplification of the materials and manufacture processes are likely to facilitate regulatory approval [22]. These aspects are particularly relevant to the manufacture of large volume scaffolds or high quantities of materials, such as those that might be required for tissue augmentation in plastic and reconstructive surgery after trauma (e.g., breast reconstruction) [23,24,25]. In these applications, it is desirable to have mechanically robust porous scaffolds that maintain their shape and volume, and guide tissue regeneration into the desired structure. This can be particularly challenging for soft tissues, as the material ideally needs to be hydrated and mimic the ECM mechanical properties, yet elastic and shape persistent to avoid fracture and throttling due to the stress caused by the collapse of the scaffold by the action of an external force. Indeed, individual cells can respond to changes in these stresses, varying from morphological alterations to changes in gene expression. 

For breast reconstruction, various porous scaffolds have been tested ranging from additive manufactured rigid plastics [25,26,27,28] (e.g., poly(glycolide-*co*-lactide) [29]) to soft hydrogels (e.g., natural biopolymers [23,28], PEG [30,31,32], and poly(acrylamide) [33]). Although rigid plastics maintain their shape and volume, the modulus of these materials and their slow degradation may not be favourable for soft tissue engineering. In comparison, hydrogels provide a more natural environment for soft tissue formation, but generally have poor mechanical strength and shape persistence. Thus, hydrogel scaffolds with good mechanical integrity and tunable degradation rates are very appealing. Whilst scaffolds could potentially be loaded with cells (e.g., adipocytes, preadipocytes, adipose-derived stem cells) before implantation [23,25,28,29,30,31,34,35], a lack of nutrient and oxygen supply to the implanted cells prior to tissue ingrowth and angiogenesis are major concerns that would result in a loss of viability, particularly for large constructs. An alternative is to implant porous scaffolds that promote tissue infiltration and growth from the host, although formation of adipose rich tissue is particularly difficult. Nevertheless, the formation of well vascularised connective tissue throughout the scaffold would provide a suitable environment for subsequent injection of autologous fat grafts, and provide structural support for the maturing tissue construct [24,25]. Regardless of the approach that is employed, the ability to tailor the mechanical properties and biodegradation rate of scaffolds are important parameters for tissue augmentation and breast reconstruction. 

Previously we fabricated highly porous and robust PEG-based sponges that showed excellent tissue responses in vivo [36]. In this case, the highly interconnected pores were generated from gas bubbles that formed due to the acid chloride/alcohol reaction used in the preparation [36]. Although this is a convenient method to introduce porosity, direct and simultaneous control over the pore size, degradation rate and mechanical properties of the sponges was difficult to achieve. Therefore, this study describes the fabrication and evaluation of porous polyester-ether hydrogels (PEH) produced via similar acid chloride/alcohol chemistry, but with significantly improved scope to control the scaffolds properties. The PEHs were prepared directly from commercially available reagents via a cost-effective, facile, and scalable manufacturing process. Furthermore, we demonstrate that the choice of cross-linker and the incorporation of a hydrophobic modifier facilitates the manufacture of hydrogels with tunable swelling, mechanical and degradation characteristics, leading to PEHs with excellent mechanical integrity under compression. In combination, these parameters provide the means to adjust the properties of the hydrogels, allowing for tissue-specific adaptation. In vivo, the PEHs display high tissue permeability and vascularization, fast biodegradation, good biocompatibility and minimal inflammatory response. The tunable properties and favourable tissue interactions of the PEHs make them promising candidates for tissue engineering applications.

## 2. Experimental Section

### 2.1. Materials

All reagents were used as received unless otherwise stated. Pentaerythritol ethoxylate (PE; *M*_n_ ~ 800 Da), poly(ethylene glycol) (PEG_600_; *M*_n_ ~ 600 Da), ε-caprolactone (97%), sebacoyl chloride (≥95%), succinyl chloride (95%), 1,3,5-benzene tricarbonyltrichloride (trimesoyl chloride) (98%), *trans*-2-[3-(4-tert-butylphenyl)-2-methyl-2-propenylidene]malononitrile (DCTB) (≥99.0%), stannous octoate (92.5–100%), 2,2′-dithiodiethanol (90%), sodium trifluoroacetate (NaTFA) (99.999%) and phosphate buffered saline (PBS) tablets were purchased from Sigma-Aldrich (St. Louis, MO, USA). Gibco^TM^ Dulbecco’s Modified Eagle Medium (DMEM), l-glutamine, trypsin-EDTA (0.05%), trypan blue (0.4%), Fetal Bovine Serum (FBS) and penicillin-streptomycin were obtained from Thermo Fisher Scientific (Waltham, MA, USA). DMEM was supplemented with 10% *v*/*v* FBS, 1% *v*/*v* l-glutamine and 1% *v*/*v* penicillin-streptomycin prior to use for the cell viability assays. Dichloromethane (≥99.5%), tetrahydrofuran (THF) (Honeywell, Morris Plains, NJ, USA, 99.99%), ethanol (undenatured 100%), sodium carbonate (≥99.2%, anhydrous), sodium chloride (≥99%) and toluene (≥99.5%) were purchased from Chem-Supply (Adelaide, Australia). CelltiterAqueousOne solution for cell viability assays was obtained from Promega (Madison, WI, USA). Anti-CD68 antibody (ED1) antibody was purchased from AbD Serotec, Oxford, UK.

### 2.2. Instrumentation

Enviro-scanning electron microscopy (E-SEM) was conducted on a FEI Quanta FEG 200 Enviro-SEM (Thermo Scientific, Waltham, MA, USA). Samples were mounted on carbon tabs. Mechanical testing was carried out using an Instron Microtester 5848 (Instron, Norwood, MA, USA) equipped with Bluehill material testing software. Matrix-assisted laser desorption ionisation time-of-flight mass spectrometry (MALDI ToF MS) was performed on a Bruker Autoflex III mass spectrometer (Bruker Daltonics, Bremen, Germany) operating in positive/linear mode. DCTB and NaTFA were dissolved in THF (10 mg/mL and 1 mg/mL, respectively) and then mixed with the polymer (1 mg/mL in THF) in a ratio of 10:1:1. An aliquot of this solution (0.3 µL) was spotted onto a ground steel target plate and the solvent allowed to evaporate. FlexAnalysis (Bruker, Bremen, Germany, version 2.2) was used to analyse the data. ^1^H NMR spectroscopy was performed on a Varian Unity400 (400 MHz) spectrometer (Agilent, Santa Clara, CA, USA), using the solvent as lock.

### 2.3. Fused Salt Template Preparation

A mortar and pestle was used to grind crystalline sodium chloride (NaCl), which was then sieved (Endecotts Ltd. (London, UK) set of standard laboratory test sieves) to obtain 300–600 µm sized salt particles. The salt particles (4 g) were transferred into polyethylene vials (28 mL) and were compressed gently with a cylindrical metal compressor. The vials were then transferred into a humidifier and maintained at room temperature (80% humidity) for 24 h to produce the fused salt templates. The fused templates were then dried *in vacuo* for 18 h (100 °C, 20 mbar), capped and placed in a desiccator until further use.

### 2.4. Synthesis of α,ω-Dihydroxyl Poly(Caprolactone) (PCL)

2,2′-Dithiodiethanol (1.08 g, 7.00 mmoL), ε-caprolactone (20.0 g, 175 mmoL) and stannous octoate (0.95 g, 2.34 mmoL) were dissolved in anhydrous toluene (45 mL) under argon and heated at 110 °C for 24 h. After cooling to room temperature, the mixture was diluted with THF (50 mL) and precipitated into cold methanol (−18 °C, 1 L). The precipitate was collected by filtration and dried *in vacuo* (0.1 mbar) to afford α,ω-dihydroxyl PCL as a white powder, 18.8 g (94%): *M*_n_ (NMR) = 3.2 kDa; *M*_n_ (MALDI ToF MS) = 3.3 kDa, Polydispersity index (PDI) = 1.07.

### 2.5. Preparation of Polyester-Ether Hydrogels (PEHs)

PEG_600_ (0.75 g, 1.26 mmoL), PE (0.50 g, 0.63 mmoL), and various amounts of dihydroxyl PCL (0 wt %, 2 wt %, or 5 wt %) were dissolved in dichloromethane (DCM, 10% *v*/*v*) (Table 1). Subsequently sebacoyl chloride (SebCl) (0.60 g, 2.51 mmoL), succinyl chloride (SucCl) (0.39 g, 2.51 mmoL), or trimesoyl chloride (TmsCl) (1.27 g, 4.77 mmoL) was added. The precursors were vortexed for 10 s and 1.2 mL of this solution was immediately pipetted into the vial containing the fused salt template and centrifuged for 30 s (4.4 krpm). The vial was then placed into an oven at 60 °C for 1 h. The cross-linked gel was removed from the vial and placed in 30 mM sodium carbonate solution (100 mL/gel). The solution was changed every 30 min for 2 h and then every hour for 3 h before a final exchange for 24 h. The resulting PEHs were then stored in PBS prior to characterisation. PEHs produced with succinyl chloride, sebacoyl chloride and trimesoyl chloride are referred to as Suc-PEH_#_, Seb-PEH_#_ and Tms-PEH_#_, respectively, whereby the subscript refers to the weight percentage (wt %) of the dihydroxyl PCL used.

### 2.6. Swelling Studies

Dehydrated PEHs (1 cm^3^) were weighed and subsequently placed in Milli-Q water for 48 h. The percentage equilibrium solvent ratio (%ESR) was calculated using the equation: %ESR = ((*W*_s_ − *W*_d_)/*W*_d_) × 100%, where *W*_s_ and *W*_d_ refer to the swollen and dried weights, respectively. The analysis was conducted in triplicate and the results averaged.

### 2.7. Pore Size Analysis Via E-SEM

PEHs (2 wt % PCL) swollen in PBS for 48 h were cut in half and mounted on a carbon tab. The exposed internal surfaces were analysed using E-SEM under low vacuum conditions to observe the porous structure of the hydrogels. ImageJ software (v1.48k, National Institute of Health, Bethesda, MD, USA) was utilised to determine the average pore sizes.

### 2.8. Compressive Evaluation of PEHs

Swollen PEHs were cut into cubes (1 cm^3^) prior to compressive testing. The PEHs were not subjected to stress preconditioning prior to compressive evaluation. The scaffolds were placed between the metal plates of an Instron Microtester 5848 (with 50 N load cell) and were subjected to compression up to 80% strain. The resulting stress versus strain profiles were used in determination of the compressive moduli of the PEHs. Some PEHs were also subjected to cyclic compression up to 80% strain to study their elastic properties.

### 2.9. In Vitro Degradation Study

PEHs (2 wt % PCL) synthesised with the three cross-linkers, were cut into cubes (5 mm^3^), dehydrated in ethanol and dried overnight in a vacuum oven (60 °C). Dried samples were weighed and placed into PBS (20 mL, 0.01% *w*/*v* sodium azide). The vials were capped and transferred to an orbital shaker (37 °C, 100 rpm). Three samples were removed from the orbital shaker at each time point (1, 2, 4 and 8 weeks) and soaked in deionised water for 30 min (3 × 20 mL). Subsequently, the hydrogels were dehydrated by soaking in ethanol for 1 h (2 × 20 mL) followed by drying *in vacuo* (60 °C, 24 h). The dried samples were then weighed and the mass values obtained were plotted against time to obtain the degradation profiles.

### 2.10. In Vitro Cytotoxicity Evaluation

For the cytotoxicity evaluation, we used a previously described method [36]. Briefly, dehydrated PEHs (100 mg) were sterilised (80% *v*/*v* ethanol solution; 30 min), washed and incubated in DMEM (37 °C, 72 h). The hydrogels were removed and the conditioned media was used in the cell viability assay. To determine the cytotoxicity of hydrogel degradation products, PEHs (500 mg) were degraded (1 M HCl, 5 mL) and the degradation products isolated via azeotropic distillation with water. For cytotoxicity studies the degradation products (100 mg) were dispersed in sterile DMEM, sterilised (UV; 30 min), and then filtered (0.22 µm). 

Confluent National Institute of Health (NIH) 3T3-L1 cells were trypsinised, diluted (1.25 × 10^5^ cells/mL) and transferred to 96 well plates (80 µL/well). The plates were placed in the incubator for 4 h, PEH conditioned media or degradation products were added, and the plates were returned to the incubator (72 h). CelltiterAqueousOne Solution was added (20 µL/well) and after 2 h in the incubator the UV/Vis absorbance was recorded at 490 and 700 nm (for background absorbance subtraction) using a Cary 50 Bio UV-Visible Spectrophotometer (Varian, Mulgrave, Australia).

### 2.11. In Vivo Implantation Study

The study was conducted in accordance with relevant national legislation on the use of animals for research. All procedures were conducted according to the guidelines of the National Health and Medical Research Council (NHMRC) of Australia and were approved by the Animal Ethics Committee, St Vincent’s Hospital, Melbourne (ID: 017/10).

Seb-PEH_2_ disks (diameter = 10 mm, height = 4 mm) were placed into plastic vials and doubly sealed in zip-lock bags prior to gamma irradiation. Gamma sterilisation was carried out at Steritech, Victoria, Australia (25 kGy minimum). Following sterilisation, the hydrogels were placed in sterile PBS for 3 h prior to implantation. The PEHs were implanted into 12 rats for 3 time points using a previously documented procedure [36], noting that for surgery, animals were anaesthetised and maintained in an anaesthetised state using isoflurane, and histology sections were stained with ED1 for detection of macrophages and foreign body giant cells. 

## 3. Results and Discussion

### 3.1. Preparation of PEHs

**Figure 1:**
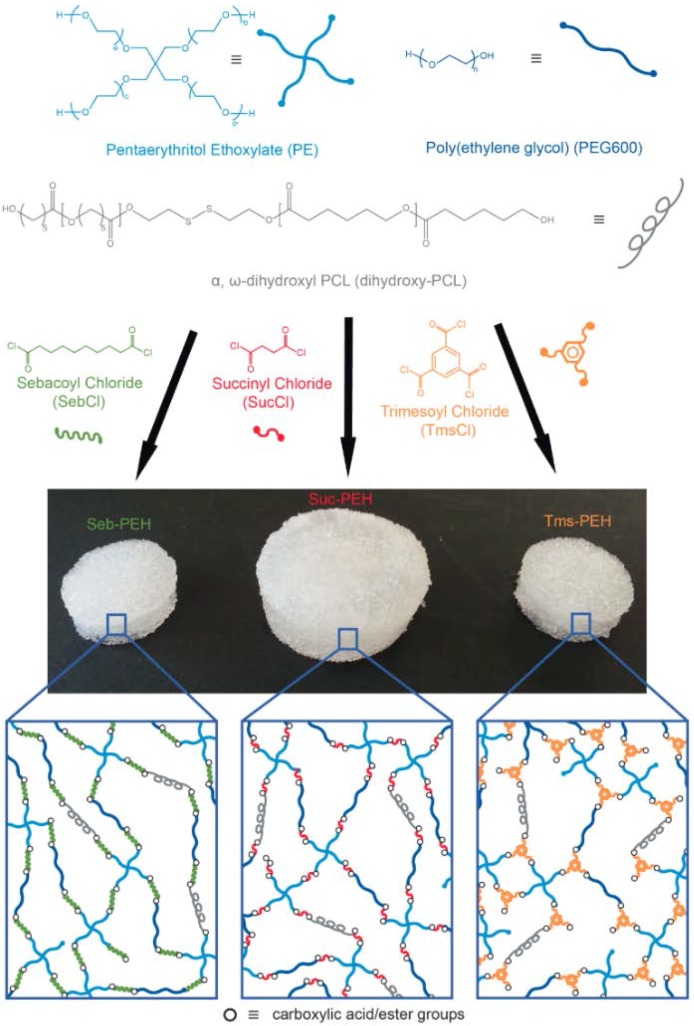
Synthesis of Suc-PEH_#_, Seb-PEH_#_, and Tms-PEH_#_ and their representative network structures.

The porous polyester-ether hydrogels (PEHs) were fabricated via a facile approach using acid chloride chemistry [36,37], whereby pentaerythritol ethoxylate (PE) and linear poly(ethylene glycol) (PEG_600_) were cross-linked with either sebacoyl chloride (SebCl), succinyl chloride (SucCl) or trimesoyl chloride (TmsCl). The precursors were dissolved in dichloromethane, combined with various amounts of α,ω-dihydroxyl poly(caprolactone) (PCL), and then allowed to gel in the presence of fused salt-templates (Figure 1). PE, a low molecular weight star polymer composed of four PEG arms, and PEG_600_ (*M*_n_ ~ 600 Da) are inexpensive and commercially available hydroxyl-terminated PEG derivatives. PEG_600_ was used to decrease the cross-linking density and stiffness of the hydrogels, as well as to slow down the cross-linking reaction so that the hydrogel precursor mixture could easy infiltrate into the template pores prior to gelation. SebCl and SucCl are diacid chlorides derived from naturally occurring carboxylic acids, whereas TmsCl is a triacid chloride derivative of benzene tricarboxylic acid. The cross-linking reaction between the acid chloride groups of SebCl, SucCl, TmsCl, and the hydroxyl end-groups of the PEG derivatives leads to the formation of an ester bonded network and the release of HCl, which is neutralised in subsequent washing steps. For all of the PEHs, the PE: acid chloride: PEG_600_ mole ratio was kept constant at 1:4:2 in order to provide the same theoretical cross-linking density for all of the hydrogels. This theoretically provides a completely cross-linked network for PEHs prepared with SebCl and SucCl. In the case of the triacid chloride TmsCl there is an excess of acid-chloride groups that during the aqueous washing steps would be hydrolysed to carboxylic acids and could potentially be used for the conjugation of bioactive molecules to modulate cell behaviour as required.

Previously, we have demonstrated that the mechanical properties of PEG-based hydrogels can be improved through the covalent incorporation of PCL [11,37]. Therefore, telechelic dihydroxyl PCL was cross-linked within the hydrogel network (Figure 1). To produce an interconnected porous structure that would allow the penetration of cells and vascularisation within the hydrogels, sacrificial fused sodium chloride templates (particle size 300–600 µm) were used. Fused salt templates were prepared by packing pre-sieved salt particles into a vial followed by exposure to a humid environment, which causes the salt particles to fuse together at points of contact. This approach ensures that the pores of the resulting hydrogels are interconnected allowing the template to be easy dissolved and washed away, and for tissue to penetrate throughout the scaffold. Following cross-linking, the PEHs were subsequently immersed in a solution of sodium carbonate to neutralise any HCl that may be trapped within. The hydrogels were referred to as X-PEH_#_, whereby the prefix denotes the cross-linker and the subscript denotes the wt % of PCL used to prepare the PEHs (e.g., Suc-PEH_2_ refers to a succinyl chloride cross-linked hydrogel with 2 wt % PCL). 

### 3.2. Swelling Characteristics of PEHs

To determine the effect of the cross-linker, and PCL content on swelling properties, the mass of the fully swollen and dried PEHs were obtained and used to calculate the percentage equilibrium swelling ratios (%ESRs) (Table 2). Out of all the PEHs prepared, Suc-PEHs clearly displayed much higher %ESRs. The longer alkyl backbone and the benzene ring of the ester cross-links generated from using SebCl and TmsCl, respectively, as well as higher molecular weight of the cross-linkers, create a more hydrophobic environment within Seb-PEHs and Tms-PEHs as compared to Suc-PEHs, and hence reduce the water absorbing capabilities of the PEHs. SucCl has a shorter alkyl backbone and hence the resulting PEHs are more hydrophilic and absorb more water. 

As the PCL content in the PEHs was increased from 0 wt % to 5 wt % there was a reduction in the %ESR, which correlated with the overall increase in the hydrophobic content wt % (Table 2). This is consistent with the effects of hydrophobicity observed for PEHs prepared using SebCl and TmsCl cross-linkers. The effect of hydrophobic components on the repulsion of water and its subsequent effects on hydrogel swelling are also supported by other studies [38].

### 3.3. Evaluation of Porous Structure

**Figure 2:**
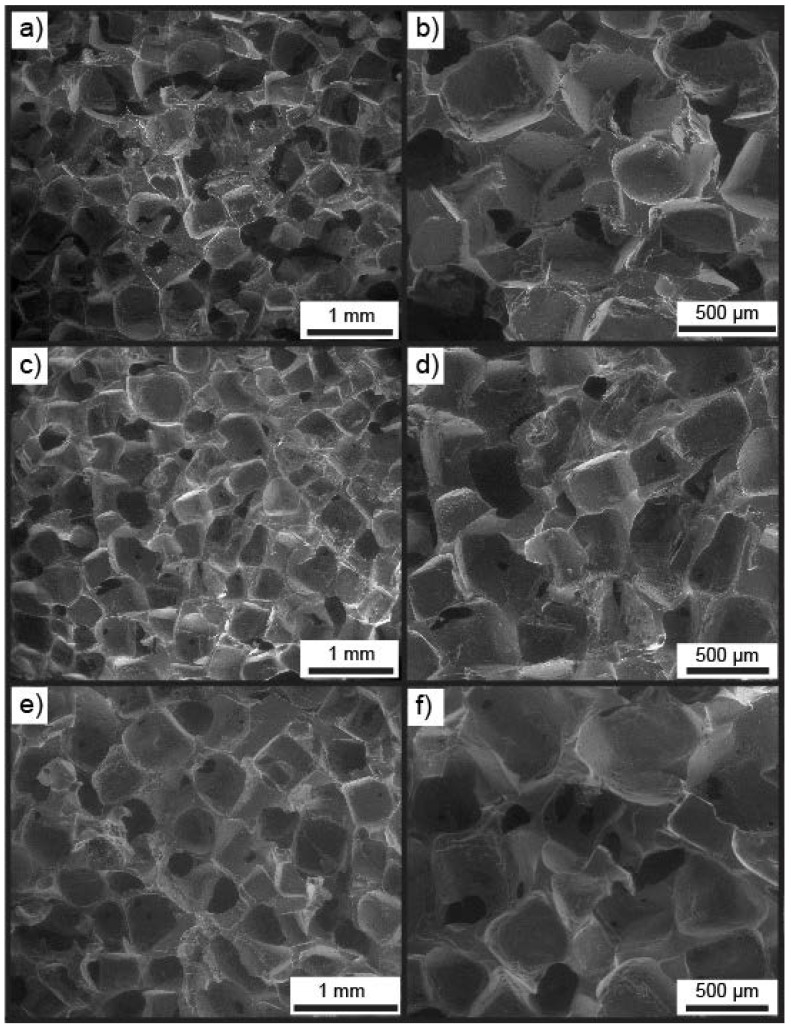
Enviro-SEM images of (**a**,**b**) Suc-PEH_2_, (**c**,**d**) Seb-PEH_2_ and (**e**,**f**) Tms-PEH_2_.

Enviro-scanning electron microscopy (ESEM) was carried out on X-PEH_2_, swollen in Milli-Q water, to determine their porous structure and the average pore size (Table 2). ESEM revealed that the pore sizes observed within the PEHs correlate well with the size of the salt particles (300–600 µm) used in the templates and were interconnected as a result of the fusing process (Figure 2). Murphy et al. has demonstrated that fusing salt particles under humid conditions leads to improved interconnectivity of the final pores [39]. The average pore sizes calculated for Suc-PEH_2_, Seb-PEH_2_, and Tms-PEH_2_ were 456, 467 and 477 µm, respectively (Table 2), which fall within the middle of the range of the salt particles. The large size and interconnected nature of the pores are anticipated to facilitate rapid tissue penetration and vascularisation as well as facile nutrient and fluid transport.

Regarding the salt-templating technique, it is possible to control the pore size of the hydrogels since the porogen particle size can be directly tailored to a specific range [40]. Importantly, this allows potential targeting of specific tissue types, as recent studies have demonstrated that certain pore sizes are better suited for the regeneration of specific tissues, including bone, skin and adipose tissue [41,42,43].

### 3.4. Mechanical Evaluation

Tissue engineering scaffolds need to maintain a 3D environment and possess suitable mechanical properties to shield the growing tissue from external forces [3,24]. Therefore, the robustness of the swollen PEHs was investigated via compressive testing with respect to the dihydroxyl PCL content and the type of cross-linker (Table 2).

**Figure 3:**
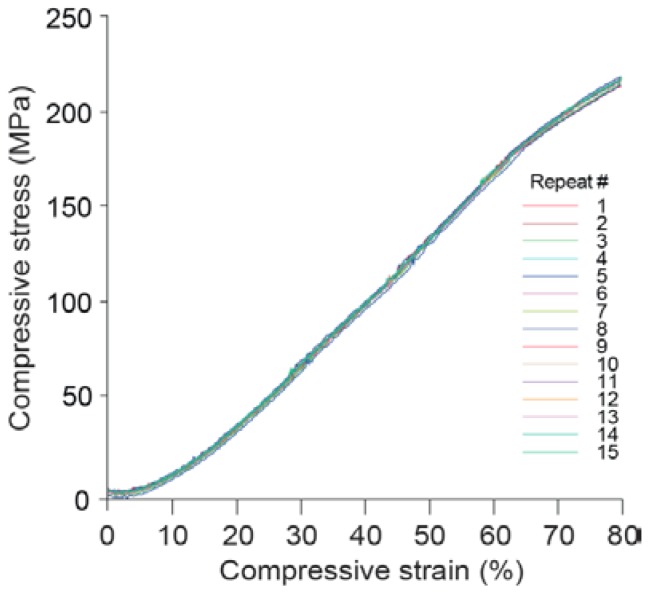
Compressive stress vs strain profile for Seb-PEH_2_ over 15 compression cycles.

Surprisingly, Seb-PEHs, Tms-PEH_2_, and Tms-PEH_5_ were found to be highly elastic, maintaining their structural integrity even after compressive strains of 80%. For example, cyclic testing of Seb-PEH_2_ demonstrated that the hydrogel remained elastic without fracture even after 15 compression cycles up to 80% (Figure 3). The compressive moduli of the Seb-PEHs increased with increasing PCL content (Table 2), which is consistent with our previous studies [11,36]. The Tms-PEHs did not initially possess the highly elastic nature of the Seb-PEHs until the incorporation of PCL. Following PCL incorporation, the Tms-PEHs also maintained an elastic structure with increasing compressive moduli with increasing PCL content (Table 2). The % compressive strain of the Suc-PEHs varied between 46% to 75%, and increased with increasing PCL content, along with the ultimate stress (Table 2). However, the compressive moduli of the Suc-PEHs remained approximately constant regardless of the PCL content.

The observed trends in the swelling of the PEHs with differing hydrophobicity could be responsible for the different compressive behaviours observed. As the Suc-PEHs possess a much higher %ESR, the polymer chains within the cross-linked network are extended and stretched, reducing their capacity to deform under compressive stress, and increasing the probability of fracture. Conversely, the Seb-PEHs and Tms-PEHs swell to a much lower degree as a result of the more hydrophobic cross-links, preventing the polymer chains from extending completely. Therefore, the Seb-PEHs and Tms-PEHs are more deformable under compressive stress, resulting in elastic behaviour even at high compressive strains. The lower elasticity observed for Tms-PEH_0_, as compared to the Seb-PEHs, is likely to result from the rigid benzoate ester cross-links as compared to the flexible alkyl esters of the latter. The incorporation of long deformable PCL chains into Tms-PEH_2_ and Tms-PEH_5_ leads to an overall increase in hydrophobicity and reduction in swelling, leading to an improvement in the elasticity.

These results demonstrate that by using different cross-linkers and varying the PCL content, the compressive properties of the PEHs can be tuned. Tuning the mechanical properties of the scaffold to match the target tissue allows improved tissue responses, especially for mechanosensitive tissue [44]. In the case of the PEHs, similar moduli are observed for tissues such as skin, kidney, and liver [45,46] thus providing access to tailored mechanical properties for specific tissues and applications. 

### 3.5. Degradation In Vitro

**Figure 4:**
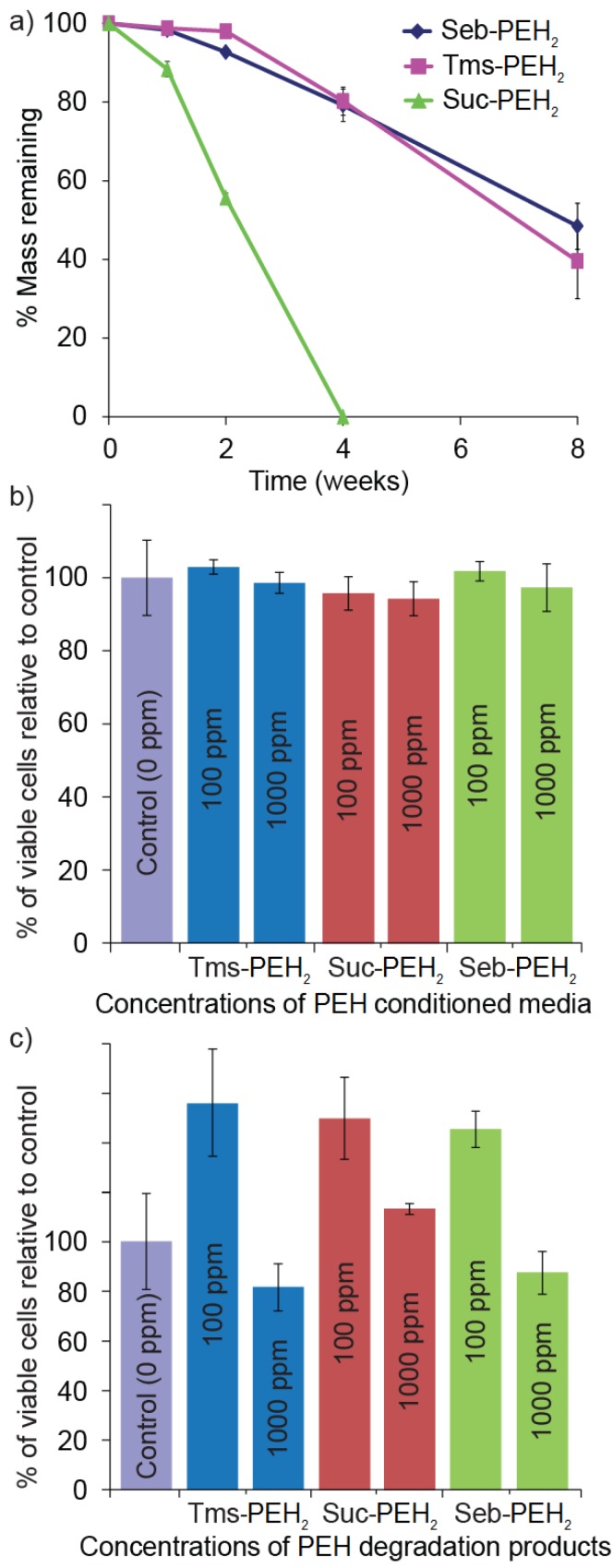
(**a**) In vitro degradation of X-PEH_2_ over 8 weeks (PBS, 37 °C). Cytotoxicity evaluation of (**b**) X-PEH_2_ conditioned media and (**c**) X-PEH_2_ degradation products.

Tissue engineering scaffolds that degrade in a controlled manner, allowing uniform tissue formation as cells regenerate within the porous structure of the scaffold are highly desirable. The advantage of the PEH system is the high density of ester cross-links, which makes them particularly susceptible to hydrolytic and enzymatic cleavage [47,48]. To determine the effect of the cross-linkers on the degradation rate, the Suc-PEH_2,_ Seb-PEH_2_, and Tms-PEH_2_ were stored in PBS solution for 8 weeks at 37 °C and the mass loss was calculated at 1, 2, 4 and 8 weeks. The degradation study revealed a mass loss of ~35%–40% in 8 weeks for Tms-PEHs and Seb-PEHs, whereas the Suc-PEHs had completely degraded by 4 weeks (Figure 4a). 

The hydrophobic cross-linkers in Seb-PEH and Tms-PEH with their long alkyl backbone (eight aliphatic carbons) and aromatic structure, respectively, are able to repel the water away from the ester cross-links more effectively than the short alkyl backbone (two aliphatic carbons) of the Suc-PEH cross-linker. Therefore, the latter is more susceptible to hydrolysis and degrades much quicker. Since the cross-linkers result in different degradation rates, this provides the possibility of utilising these three cross-linkers in combination for tuning of the degradation rates (and mechanical properties) as required. The degradation profiles obtained from this study are only representative of in vitro conditions and these rates could be further accelerated by the presence of hydrolytic enzymes in vivo. The results of the in vitro degradation study demonstrate the potential of the PEHs as biodegradable implants. A degradable implant would allow the tissue to regenerate to form a uniform structure and prevent foreign material from interfering with tissue function.

### 3.6. In Vitro Cell Viability

As implantable scaffolds, the PEHs and their degradation products need to be non-toxic. Therefore, in vitro cytotoxicity studies were conducted with 3T3 fibroblasts. The cells were incubated in the presence of PEH conditioned media at concentrations of 100 and 1000 ppm for 72 h. Regardless of the PEHs employed, minimal effect on cell viability was observed (Figure 4b). Subsequently, the cells were incubated for 72 h with various concentrations of PEH degradation products obtained from accelerated acid catalysed degradation. In the presence of 100 ppm of the degradation products a significant increase in the metabolic activity of the cells was observed (Figure 4c). For 1000 ppm of the degradation products a slight reduction in metabolic activity was observed, but relative to the control this was statistically insignificant (*p* > 0.05). 

Complete hydrolytic degradation of the ester bonds in the PEHs theoretically affords low toxicity compounds, including PEG derivatives, sebacic acid, succinic acid, trimesic acid, and 6-hydroxyhexanoic acid (from complete degradation of the PCL). PEG is considered to be non-toxic and is approved by the FDA for cosmetic and drug delivery applications [49,50,51]. Sebacic acid is an intermediate in fatty acid oxidation [52]. Succinic acid is also naturally present in cells and plays a significant role in the citric acid cycle [53]. Trimesic acid, a synthetic tricarboxylic acid, and 6-hydroxyhexanoic acid have high lethal dose (LD_50_) values of 8.4 and 4.3 g/kg, respectively, which are unlikely to be reached through degradation of the scaffolds [54]. In vivo, degradation events are also likely to produce polymeric fragments that are cleared from the body. In particular, PCL degrades to low molecular weight fragments in vivo that can be completely excreted [55]. As the Seb-PEH_2_ conditioned media and degradation products had minimal effect on the metabolic activity of cells in vitro, and Seb-PEH_2_ displayed excellent elastic properties, it was selected for further studies in vivo.

### 3.7. In Vivo Assessment

**Figure 5:**
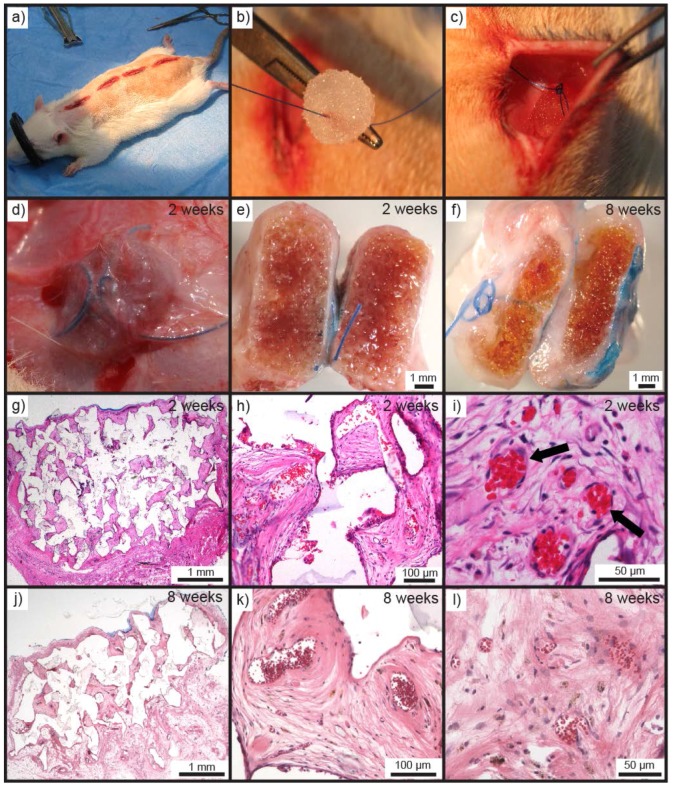
(**a**) Prepared subcutaneous pockets for implantation of Seb-PEH_2_. (**b**) Seb-PEH_2_ cylinders prior to implantation and suturing. (**c**) Seb-PEH_2_ inserted and sutured into the dorsal pocket. (**d**) Seb-PEH_2_ prior to removal at 2 weeks. Macroscopic cross-sections of Seb-PEH_2_ explants at (**e**) 2 and (**f**) 8 weeks. H&E stained sections of Seb-PEH_2_ explants removed at 2 weeks at (**g**) 1.25×, (**h**) 10×, and (**i**) 20× magnification (note black arrows marking the central vasculature containing strongly eosinophilic red blood cells). H&E stained sections of Seb-PEH_2_ explants removed at 8 weeks at (**j**) 1.25×, (**k**) 10×, and (**l**) 20× magnification.

To assess the biocompatibility, biodegradation, and immune response to the PEHs in vivo, as well as tissue in-growth and vascularisation, Seb-PEH_2_ was implanted in rats [11,36]. Seb-PEH_2_ cylinders (10 mm diameter, 4 mm height) were implanted subcutaneously in the dorsal region of male Sprague-Dawley rats (Figure 5a–c), and subsequently removed after periods of 2, 8 and 16 weeks (*n* = 4 for each time point). Removal of all of the PEHs was unremarkable without any macroscopic evidence of inflammation, toxicity or adverse effects in the surrounding tissue.

Two week samples revealed that the PEHs were well integrated into the surrounding tissue (Figure 5d). Macroscopic observation of the hydrogels following bisection revealed that the hydrogels were intact and similarly sized to when implanted (Figure 5e). The hydrogels were sectioned following fixing in paraffin and subsequently stained with H&E to observe cellular infiltration (Figure 5g–i). H&E stained sections showed the penetration of dense cellular and highly vascularised tissue into the hydrogel from the surrounding tissue. Eosinophilic red blood cells within vasculature indicated vascularisation of the constructs (Figure 5h,i). Fragments of the PEHs could also be observed between the stained tissues indicating the location of the walls of the pores of the hydrogels (Figure 5g,h). The large gaps observed in the stained sections are likely to result from dehydration and shrinkage of the PEH pore walls and the tissue during processing into paraffin.

Bisection of the 8 week harvested tissue showed a slight decrease in thickness of the size of the explant and a yellowing of the hydrogel matrix. This reduction in size and yellowing could be indicative of degradative processes. Infiltration of tissue to the centre of the scaffolds was confirmed via H&E staining, but a significant difference in the amount of penetrated tissue was not observed (Figure 5j). Although, it was noted that the tissue present after 8 weeks was of the loose-connective type, rather than the densely cellular tissue present at 2 weeks (Figure 5k,l). Vascularisation of scaffolds is crucial for the growth and survival of regenerating tissue [56,57]. Penetration of vascularised tissue to the centre of the hydrogels demonstrates the interconnected nature of the pores of the PEHs. The tissue developed within the PEH pores must have infiltrated from the surrounding tissue, since no cells were incorporated.

**Figure 6:**
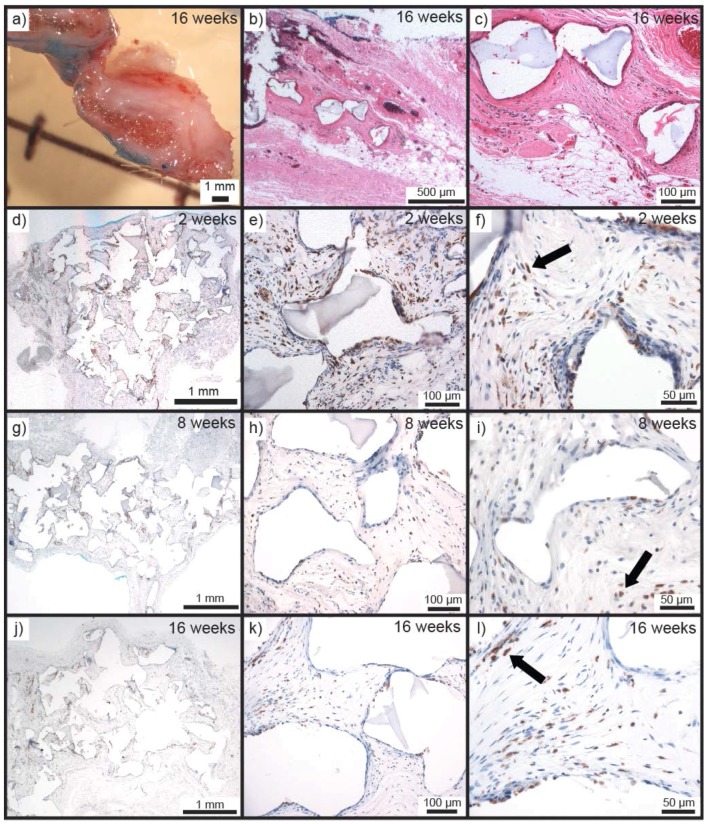
(**a**) Macroscopic cross-section of Seb-PEH_2_ removed at 16 weeks. H&E stained section of tissue where Seb-PEH_2_ was implanted (note minimal PEH remnants were observed) at (**b**) 1.25× and (**c**) 10× magnification. ED1 stained sections of Seb-PEH_2_ removed at 2 weeks at (**d**) 1.25×, (**e**) 10×, and (**f**) 20× magnification. ED1 stained sections of Seb-PEH_2_ removed at 8 weeks at (**g**) 1.25×, (**h**) 10×, and (**i**) 20× magnification (note the reduced macrophage numbers indicated by reduced staining at 8 weeks compared to 2 weeks). ED1 stained section of tissue in the Seb-PEH_2_ implantation site at 16 weeks at (**j**) 1.25×, (**k**) 10×, and (**l**) 20× magnification. (ED1 stained macrophages are indicated by arrows).

It was much more difficult to discern the implants in situ at 16 weeks compared to 2 and 8 weeks due to a marked reduction in height and greater integration into the underlying tissue. As per 2 and 8 weeks no macroscopic evidence of adverse effects was present in the tissue surrounding the anchoring suture, which was immediately dissected and processed for histology. Bisection of the harvested tissue revealed that the implants were much smaller in height and a very small amount of scaffold material was remaining (Figure 6a). H&E staining was carried out to discern the tissue morphology and observe the presence of remaining scaffold material. Analysis of the sections revealed that by 16 weeks, there was a small amount of scaffold material remaining (Figure 6b,c). Remnants were reduced in both size and number, and the tissue surrounding these was fibro-vascular tissue. This indicates that in 16 weeks the PEHs undergo major degradation, demonstrating their in vivo biodegradability.

As a response to both the tissue trauma of the implantation procedure and the presence of a hypoxic scaffold/tissue space, macrophages would be expected to be present in and around the implanted PEHs. As a means of determining macrophage and foreign body giant cell (FBGC) responses, ED1 immunostaining was carried out on all sections (Figure 6d–l) [58]. ED1 staining of 2 week samples revealed the presence of macrophages as expected. Macrophages were present mainly as well-dispersed cells within the penetrated tissue centrally within the scaffold pores, with only a few located at the tissue-hydrogel interface; in fact, the majority of cells at the interface were negative for ED1 labelling (Figure 6d–f). 

Viewing of the 8 weeks sections showed that the macrophage response was clearly diminished when compared with the 2 week time point; they were smaller in size and less numerous (Figure 6g–i). At the scaffold surface there were still minimal macrophage numbers and there was no tendency towards macrophage aggregation or FBGC formation, demonstrating the minimal response towards the hydrogel material. By 16 weeks the PEHs had undergone major degradation, as evidenced by the very small amount of scaffold and number of void spaces remaining in the sections. ED1-stained 16 weeks sections showed a very similar appearance with respect to macrophages and FBGCs to that seen at 8 weeks (Figure 6j–l). Analysis of the sections via ED1 staining clearly demonstrates that the PEHs initially resulted in a minor inflammatory response only and that this response diminished over the 16 week time course of the study, despite the continued presence of scaffold material and release of degradation products.

## 4. Conclusions

Porous polyester-ether hydrogels (PEHs) with tunable properties were produced as porous tissue engineering scaffolds using a facile, rapid, and scalable synthetic approach. A salt leaching technique was utilized to control pore sizes. Three acid chloride cross-linkers, sebacoyl, succinyl, and trimesoyl chlorides were used for hydrogel fabrication. Use of different cross-linkers allowed the fabrication of hydrogels with different swelling, mechanical, and degradation properties. The covalent incorporation of various amounts of PCL was also employed to tailor the compressive and swelling capabilities of the PEHs. In combination, these parameters provide the means to tune the swelling, degradation rates, and mechanical properties of the hydrogels allowing specific tailoring of scaffolds for various tissue types. *In vitro* degradation studies and implantation in vivo demonstrated the biodegradability of the PEHs, with major degradation over 16 weeks for implanted Seb-PEHs. *In vitro* studies using PEH conditioned media and PEH degradation products revealed negligible effect on cell proliferation at low and high concentrations. *In vivo*, no toxicity or adverse effects were observed and the rapid penetration of vascular tissue to the centre of the hydrogels was observed within 2 weeks. ED1 staining for macrophages revealed that the Seb-PEHs incite minimal inflammatory responses that are reduced from 2 weeks onwards, and are minimised by 8 weeks. Even after major degradation at 16 weeks, no adverse effects from the PEH degradation products were noted, as indicated by the minimal presence of macrophages. The use of commercially available and cheap precursors also demonstrates the PEHs’ suitability for large scale production. With desirable biodegradable, biocompatibility, and minimal inflammatory response, as well as high permeability to tissue and vascularisation, the PEHs are promising candidates for tissue augmentation and breast reconstruction. 

## Figures and Tables

**Table 1:** Reagent quantities for the preparation of Suc-PEHs, Seb-PEHs, and Tms-PEHs.

Reagent	Suc-PEH_#_	Seb-PEH_#_	Tms-PEH_#_
PE	0.50 g, 0.63 mmoL	0.50 g, 0.63 mmoL	0.50 g, 0.63 mmoL
PEG_600_	0.75 g, 1.26 mmoL	0.75 g, 1.26 mmoL	0.75 g, 1.26 mmoL
Dihydroxyl PCL (0 wt %, 2 wt %, 5 wt %)	0 mg, 33 mg, 82 mg	0 mg, 37 mg, 93 mg	0 mg, 38 mg, 96 mg
DCM (10% *v*/*v*)	151 µL	180 µL	170 µL
SucCl	0.39 g, 2.51 mmoL	-	-
SebCl	-	0.60 g, 2.51 mmol	-
TmsCl	-	-	0.67 g, 2.51 mmoL

**Table 2:** Percentage equilibrium swelling ratio (%ESR), weight percentage of hydrophobic content, average pore size and mechanical characteristics (under compression testing) for the PEHs.

PEH ^a^	%ESR	Hydrophobic Content (wt %) ^b^	Average Pore Size (µm)	Ultimate Stress (MPa)	Ultimate Compression (%)	Compressive Modulus (KPa)
*Suc-PEH*_0_	1209 ± 65	24	-	33 ± 3.4	46 ± 2.0	81.0 ± 11
*Suc-PEH*_2_	1184 ± 56	25	456 ± 114	51 ± 5.3	58 ± 2.1	89.0 ± 0.1
*Suc-PEH*_5_	1139 ± 52	27	-	63 ± 4.2	75 ± 4.1	81.0 ± 10
*Seb-PEH*_0_	327 ± 4.5	32	-	- ^c^	- ^c^	183 ± 28
*Seb-PEH*_2_	310 ± 2.1	34	467 ± 118	- ^c^	- ^c^	250 ± 20
*Seb-PEH*_5_	301 ± 2.3	36	-	- ^c^	- ^c^	330 ± 5.7
*Tms-PEH*_0_	370 ± 6.3	35	-	124 ± 18	60 ± 9.2	192 ± 6.0
*Tms-PEH*_2_	340 ± 0.9	35	477 ± 102	- ^c^	- ^c^	148 ± 3.0
*Tms-PEH*_5_	321 ± 5.5	38	-	- ^c^	- ^c^	193 ± 6.0

^a^ Prefix denotes the cross-linker and subscript denotes the wt % of PCL used to prepare the polyester-ether hydrogels. ^b^ Determined from the mass of the cross-linker and PCL used in the formulations. ^c^ No fracture or defects were observed up to the maximum compressive strain of 80% used in this study.
